# Disease Classification in Hansaraj Nidan and Madhava Nidana: A Comparative Critical Review

**DOI:** 10.7759/cureus.117311

**Published:** 2026-10-01

**Authors:** Yogita Jain, Sumant Moreshwar Pande

**Affiliations:** 1 Department of Rog Nidan, Mahatma Gandhi Ayurved College, Hospital and Research Centre, Wardha, IND

**Keywords:** ayurvedic nosology, disease classification, hansaraj nidan, madhava nidana, nidana panchaka, textual analysis

## Abstract

Ayurvedic nosology has developed from the classical compendia into specialized diagnostic texts. Madhava Nidana is an influential and systematic text on disease diagnosis, whereas Hansaraj Nidan is a later and comparatively unexplored diagnostic work. This review compares the historical organization, disease classification, diagnostic components, disease subtypes, complications, and prognostic descriptions of the two texts. A comparative critical textual analysis was conducted using authenticated editions and recognized commentaries, with descriptive quantitative content mapping. The five components of the Nidana Panchaka - Nidana (causative factors), Purvarupa (prodromal features), Rupa (established clinical features), Upashaya-Anupashaya (relieving and aggravating factors), and Samprapti (pathogenesis) - along with Bheda (disease subtypes), Upadrava (complications), and prognosis were systematically extracted for each disease record. Common, unique, and differently classified disease entities were identified, and a diagnostic-completeness score was calculated based on the explicit documentation of these components, with interpretations reviewed by subject experts in Ayurveda. The two texts showed substantial overlap but differed in chapter structure, terminology, disease subdivision, and diagnostic emphasis. Hansaraj Nidan presented distinctive variants of Jwara (febrile disorders), Hikka (hiccup), Shwasa (breathing disorders), Udavarta (a disorder associated with upward movement of bodily urges), and ophthalmic diseases, whereas Madhava Nidana documented pathogenesis, complications, and prognosis more frequently across the records assessed. Rupa and Nidana were the most consistently documented diagnostic components in both texts. Both texts contribute substantially to Ayurvedic disease classification, and comparative mapping highlights their shared terminology, diagnostic distinctions, and changes in disease classification. The findings are descriptive and reflect the documentation content of the editions examined.

## Introduction and background

Disease classification is the foundation of clinical study in Ayurveda, providing systematic knowledge of etiology, pathogenesis, clinical presentation, prognosis, and therapy. Classical Ayurvedic texts describe diseases as innumerable and classify them on several grounds, including dosha (fundamental regulatory principles of the body), dushya (body tissues and components affected by disease), srotas (physiological channels or pathways), causative factors, anatomical site, mode of onset, severity, and prognosis. The Charaka Samhita presents a holistic approach to disease through Hetu (etiological factors), Linga (clinical signs and symptoms), and Aushadha (therapeutic measures), and situates physical and psychological disorders within a personalized clinical context [1]. The Sushruta Samhita organizes disease within surgical, anatomical, traumatic, and systemic frameworks [2], while the Ashtanga Hridaya describes disease development through Sankhya (numerical classification), Vikalpa (variation or subdivision), Pradhanya (predominance), Bala (strength or severity), and Kala (time or temporal factors), incorporating these principles into Ayurvedic disease classification [3]. Comparison of the Charaka Nidana Sthana (the section dealing primarily with disease diagnosis) with later classical texts shows that, although core diagnostic principles were retained, disease terminology changed, complications were elaborated, and the number of described diseases increased over time [4].

In this tradition, Roga denotes a state of pain, dysfunction, or suffering, while Roga Nidana refers to the systematic study of the factors and clinical features associated with disease. The principal diagnostic framework is the Nidana Panchaka, meaning the five-part approach to disease diagnosis: Nidana (causative factors), Purvarupa (prodromal features), Rupa (established clinical features), Upashaya-Anupashaya (relieving and aggravating factors), and Samprapti (pathogenesis). These components, used together or selectively, support early diagnosis, differentiation of clinically similar conditions, assessment of disease progression, and selection of therapy [5].

Madhava Nidana, also known as Rugvinishchaya, is a landmark text in Ayurvedic diagnostic literature. It arranges diseases according to their clinical presentation and describes their etiology, prodromal features, symptoms, pathogenesis, varieties, complications, and prognosis in a clinically oriented manner [6]. Madhavakara systematically reclassified diseases, granted independent status to conditions previously regarded as subsidiary, and introduced new clinical entities [7]. In doing so, the text organizes disease along the major clinical lines of Ayurveda and assigns importance to disorders formerly treated only as symptoms or secondary conditions [3].

Hansaraj Nidan, also titled Bhishak Chakra Chittotsava, is a later diagnostic work focused on Roga Nidana, or the systematic study of disease causation and clinical features. It is preserved in manuscript copies in several Indian institutions [8]. Generally assigned to the 15th-17th century CE, it acknowledges earlier authorities, including Charaka, Sushruta, Vagbhata, and Madhavakara, and therefore represents a synthesis of earlier diagnostic traditions while offering distinctive descriptions and variants of diseases, such as Jwara (febrile disorders), Hikka (hiccup), Shwasa (breathing disorders), Udavarta (a disorder associated with upward movement of bodily urges), and ophthalmic disorders [8]. A preliminary comparison indicates that Hansaraj Nidan and Madhava Nidana share considerable disease material while differing in emphasis, arrangement, and interpretation of clinical features and complications [8].

Comparative textual studies have previously examined chapter sequence, terminology, and diagnostic content across Ayurvedic works. For example, a comparison of Vaidyachandrodaya with Madhava Nidana documented additions, omissions, and rearrangement of chapters alongside differences in diagnostic detail [9]. However, earlier work has largely analyzed individual disorders in isolation, and a comprehensive mapping of the classification systems of Hansaraj Nidan and Madhava Nidana is lacking. The present review therefore compares the historical organization, disease categories, diagnostic components, disease types and subtypes, complications, and prognostic classifications of the two texts; identifies common, unique, and differently classified diseases; evaluates the relative completeness of their diagnostic descriptions; and considers the contribution of both works to the evolution and current interpretation of Ayurvedic nosology.

## Review

Selection of texts

Study Design and Selection of Texts

This was a comparative, critical textual analysis with descriptive quantitative content mapping of disease classification in Hansaraj Nidan and Madhava Nidana. The primary sources were the Sanskrit text and English translation of Hansaraj Nidan (Bhishak Chakra Chittotsava) and the Madhava Nidana together with its Madhukosha and Atanka Darpana commentaries. These works were examined for terminology, the evolution of diagnostic concepts, and differences in interpretation. The spelling "Hansaraj Nidan" is used throughout this manuscript for consistency; the same work appears in the reference list under the transliteration used by its respective publishers, including "Hansaraja Nidana" and "Hamsaraja Nidana."

Eligibility Criteria and Source Selection

Eligible sources were primary verses, authentic commentaries, authenticated translations, and peer-reviewed comparative textual studies that specifically addressed Roga Nidana, disease classification, the Nidana Panchaka, or comparative Ayurvedic textual analysis. Sources were drawn from institutional libraries, recognized Ayurvedic databases, and scholarly search engines. Works lacking clear authorship, reliable textual attribution, or complete bibliographic data were excluded, as were duplicate publications, non-scholarly web pages, and interpretations not grounded in classical sources.

Secondary literature was identified through searches of relevant scholarly databases and Ayurvedic literature resources using combinations of the terms "Hansaraj Nidan", "Hamsaraja Nidana", "Madhava Nidana", "Roga Nidana", "Nidana Panchaka", "disease classification", and "Ayurvedic nosology". Reference lists of relevant publications were also screened to identify additional comparative and historical sources.

Data extraction and disease mapping

A structured data-extraction sheet recorded, for each disease, its Sanskrit name, a standardized name, the clinical category, the basis of classification, and the components Nidana, Purvarupa, Rupa, Upashaya-Anupashaya, Samprapti, Bheda (subtypes), Upadrava (complications), and prognosis. Each distinctly named disease described in a text was treated as one disease record; on this basis, 80 records were extracted from Hansaraj Nidan and 79 from Madhava Nidana. The details are provided in Appendix 1. Records that referred to the same clinical condition across the two texts, whether under an identical term or a recognized synonym, were mapped to a single standardized disease entity, and subtypes (Bheda) were catalogued under their parent disease rather than counted as separate entities. Synonymy or equivalence was determined from the textual description, commentary, and expert consensus. Merging the two record sets under these standardized entities produced 68 entities common to both texts, 12 confined to Hansaraj Nidan, and 11 confined to Madhava Nidana, giving 91 distinct entities in the combined corpus (68 + 12 + 11 = 91; equivalently, 68 + 12 = 80 Hansaraj Nidan records and 68 + 11 = 79 Madhava Nidana records). Entities present in both texts were further classified as similarly or differently classified according to their terminology, category placement, and number of subtypes. Disease grouping, terminology, and the emphasis placed on subtypes and diagnostic detail were also noted.

Comparative classification and diagnostic scoring

The distribution of diseases, the principles of classification, and the diagnostic components, complications, and prognostic descriptions of the two texts were compared. For each disease record, eight diagnostic components were assessed: the five elements of the Nidana Panchaka (Nidana, Purvarupa, Rupa, Upashaya-Anupashaya, and Samprapti) together with disease subtypes (Bheda), complications (Upadrava), and prognosis. Each component was scored dichotomously as documented (1) when the text described it explicitly and not documented (0) when it was absent. A component that was only alluded to, or that was too ambiguous to attribute with confidence, was scored as not documented rather than assumed present, and such instances were flagged for expert review. The diagnostic-completeness score for a record was the number of documented components divided by the eight assessed components, expressed as a percentage, and record-level scores were averaged for each text.

Diagnostic-completeness score (%) = (documented components / 8 assessed components) × 100

Findings were summarized using descriptive counts, percentages, and comparison matrices; no inferential statistical testing was undertaken. The diagnostic-completeness score was based on the binary presence or absence of each predefined diagnostic component. Thus, the metric assessed whether a component was explicitly documented rather than the qualitative depth or extent of its description; a brief mention and a detailed multi-verse description were assigned the same score.

Validation and provenance of source materials

Data extraction was carried out using the structured sheet, and the extracted entries were checked against the selected editions at the verse level. Sanskrit terminology and disease interpretation were reviewed in consultation with subject experts in Samhita Siddhanta (the study and interpretation of classical Ayurvedic texts and principles) and Roga Nidana, and discrepancies were resolved by re-examination of the source text and discussion until consensus was reached. This comparative textual analysis was undertaken as part of a doctoral research project registered with the Institutional Ethics Committee of Mahatma Gandhi Ayurved College, Hospital and Research Centre, Salod (H), Wardha (approval ref. MGACHRC/IEC/Oct-2024/882, issued October 9, 2024, for the project “Comparative Critical Analysis of Hansaraj Nidan with Madhav Nidan”). The analysis was confined to published classical texts, commentaries, and translations; it did not involve human participants, patient records, or clinical data.

Historical, textual, and structural characteristics

Both works are specialized Ayurvedic texts centered on Roga Nidana. Madhava Nidana is the earlier text and is arranged in systematic, disease-specific chapters that follow the broad clinical branches of Ayurveda, whereas Hansaraj Nidan (Bhishak Chakra Chittotsava) belongs to a later compilatory tradition; it draws on earlier compendia but adds a number of distinctive disease names and subdivisions. Both are written in Sanskrit and arranged in a disease-oriented manner, yet they differ in chapter order, terminology, disease subdivision, and diagnostic emphasis. Madhava Nidana carries a broad commentarial tradition (Madhukosha and Atanka Darpana), whereas the organization of Hansaraj Nidan varies across its manuscripts and printed editions. The principal historical and structural features, including the major traditional exegetical commentary associated with each text, are summarized in Table 1.

**Table 1:** Comparative historical, textual, and structural characteristics of Hansaraj Nidan and Madhava Nidana Source: Data for historical, textual, and structural characteristics were derived from the selected editions and relevant secondary literature [6,7,10]. Disease-record counts represent the authors' systematic textual extraction. Table created by the authors based on the data extracted and analyzed in this review; not reproduced or adapted from an external source. CE: Common Era

Characteristic	Hansaraj Nidan	Madhava Nidana
Author	Acharya Hansaraja	Acharya Madhavakara
Alternative title	Bhishak Chakra Chittotsava	Rugvinishchaya
Approximate period	15th–17th century CE	7th–8th century CE
Primary subject	Disease diagnosis and classification	Disease diagnosis and pathology
Language	Sanskrit	Sanskrit
Organization	Disease-oriented compilatory arrangement	Systematic disease-specific chapters
Major commentary (principal traditional exegesis)	Hansarajarthabodhini (selected editions)	Madhukosha and Atanka Darpana
Disease records extracted	80	79

Category-wise distribution and classification of diseases

Systematic textual extraction identified 80 disease records in Hansaraj Nidan and 79 in Madhava Nidana. Gastrointestinal disorders formed the largest category in both texts (16 and 17 records, respectively). Febrile (Jwara) classifications were more numerous in Hansaraj Nidan (12) than in Madhava Nidana (8), reflecting greater subdivision of Jwara in the former. The respiratory, cardiovascular, neurological, urinary, dermatological, musculoskeletal, reproductive, and pediatric categories were similarly distributed, while metabolic and toxicological conditions were slightly more numerous in Madhava Nidana. Overall coverage of the major clinical categories was broadly comparable, with the main differences arising in disease subdivision and category placement. The category-wise distribution is shown in Table 2.

**Table 2:** Category-wise mapping and distribution of diseases described in Hansaraj Nidan and Madhava Nidana Source: Authors' systematic textual extraction and classification based on the selected editions of Hansaraj Nidan and Madhava Nidana [6,10]. Table created by the authors based on the data extracted and analyzed in this review; not reproduced or adapted from an external source. ENT: ear, nose, and throat

Disease category	Hansaraj Nidan, n	Madhava Nidana, n	Comparative observation
Jwara disorders (febrile conditions)	12	8	Greater subdivision in Hansaraj
Gastrointestinal	16	17	Comparable coverage
Respiratory	7	7	Similar distribution
Cardiovascular	5	5	Similar major classification
Neurological	6	6	Comparable coverage
Urinary	5	5	Similar distribution
Metabolic	5	6	Slightly higher in Madhava
Dermatological	8	8	Comparable coverage
Musculoskeletal	5	5	Similar distribution
Reproductive	4	4	Comparable coverage
Pediatric	3	3	Similar distribution
ENT and ophthalmic	3	3	Differences in subdivisions
Toxicological	1	2	Higher in Madhava
Total	80	79	—

Common, unique, and differently classified diseases

Sixty-eight disease entities were described in both texts: 53 with broadly similar classification and 15 differing in terminology, chapter placement, number of subtypes, or diagnostic presentation. Twelve entities were unique to Hansaraj Nidan-including distinct subdivisions of Jwara, age-related Hikka, Atishwasa (excessive or labored breathing), Udavarta, and selected ophthalmic disorders-and 11 were unique to Madhava Nidana. The combined corpus therefore comprised 91 distinct disease entities, indicating substantial conceptual continuity together with meaningful divergence between the two texts. These entities are summarized in Table 3.

**Table 3:** Common, unique, and differently classified diseases in Hansaraj Nidan and Madhava Nidana Table created by the authors based on the data extracted and analyzed in this review; not reproduced or adapted from an external source.

Comparison category	Number of entities	Percentage of 91 entities
Common and similarly classified	53	58.2%
Common but differently classified	15	16.5%
Unique to Hansaraj Nidan	12	13.2%
Unique to Madhava Nidana	11	12.1%
Total distinct entities	91	100.0%

Nidana Panchaka components and disease subtypes

Rupa was the most consistently documented component (78 of 80 Hansaraj records and all 79 Madhava records), followed by Nidana (72 and 75 records, respectively). Purvarupa, Upashaya-Anupashaya, and Samprapti were comparatively better documented in Madhava Nidana, and Upashaya-Anupashaya was the least frequently documented component in both texts (31 Hansaraj and 38 Madhava records). Clinical symptoms and signs were well represented in both works, while Madhava Nidana provided comparatively detailed etiopathology and a greater number of disease subtypes, complications, and prognostic classifications. A few disorders, however, showed subdivisions unique to Hansaraj Nidan. These findings are presented in Tables 4-5.

**Table 4:** Comparative documentation of Nidana, Purvarupa, Rupa, Upashaya–Anupashaya, and Samprapti Table created by the authors, based on the structured data-extraction sheet described in the Review section; not reproduced or adapted from an external source.

Diagnostic component	Hansaraj Nidan, n (%) (N = 80)	Madhava Nidana, n (%) (N = 79)
Nidana	72 (90.0%)	75 (94.9%)
Purvarupa	49 (61.3%)	56 (70.9%)
Rupa	78 (97.5%)	79 (100.0%)
Upashaya–Anupashaya	31 (38.8%)	38 (48.1%)
Samprapti	58 (72.5%)	66 (83.5%)

**Table 5:** Comparison of disease types, subtypes, complications, and prognostic classifications Table created by the authors based on the data extracted and analyzed in this review; not reproduced or adapted from an external source.

Diagnostic feature	Hansaraj Nidan, n (%) (N = 80)	Madhava Nidana, n (%) (N = 79)
Disease types or subtypes	65 (81.3%)	70 (88.6%)
Upadrava or complications	44 (55.0%)	52 (65.8%)
Prognostic classification	41 (51.3%)	49 (62.0%)

Diagnostic-component documentation

In the diagnostic-completeness assessment, Hansaraj Nidan and Madhava Nidana had mean scores of 68.4% and 76.7%, respectively. The 8.3-percentage-point difference reflected the more frequent documentation of Purvarupa, Upashaya-Anupashaya, Samprapti, complications, and prognosis in Madhava Nidana. Both texts showed high documentation frequencies for Rupa and Nidana, consistent with their diagnostic focus, and Hansaraj Nidan included distinctive subdivisions of febrile disorders and several uncommon clinical forms. Overall, Madhava Nidana documented the assessed components more frequently, whereas Hansaraj Nidan showed substantial documentation of the core diagnostic components, together with additional subdivisions and distinctive terminology. The scores are plotted in Figure 1.

**Figure 1:**
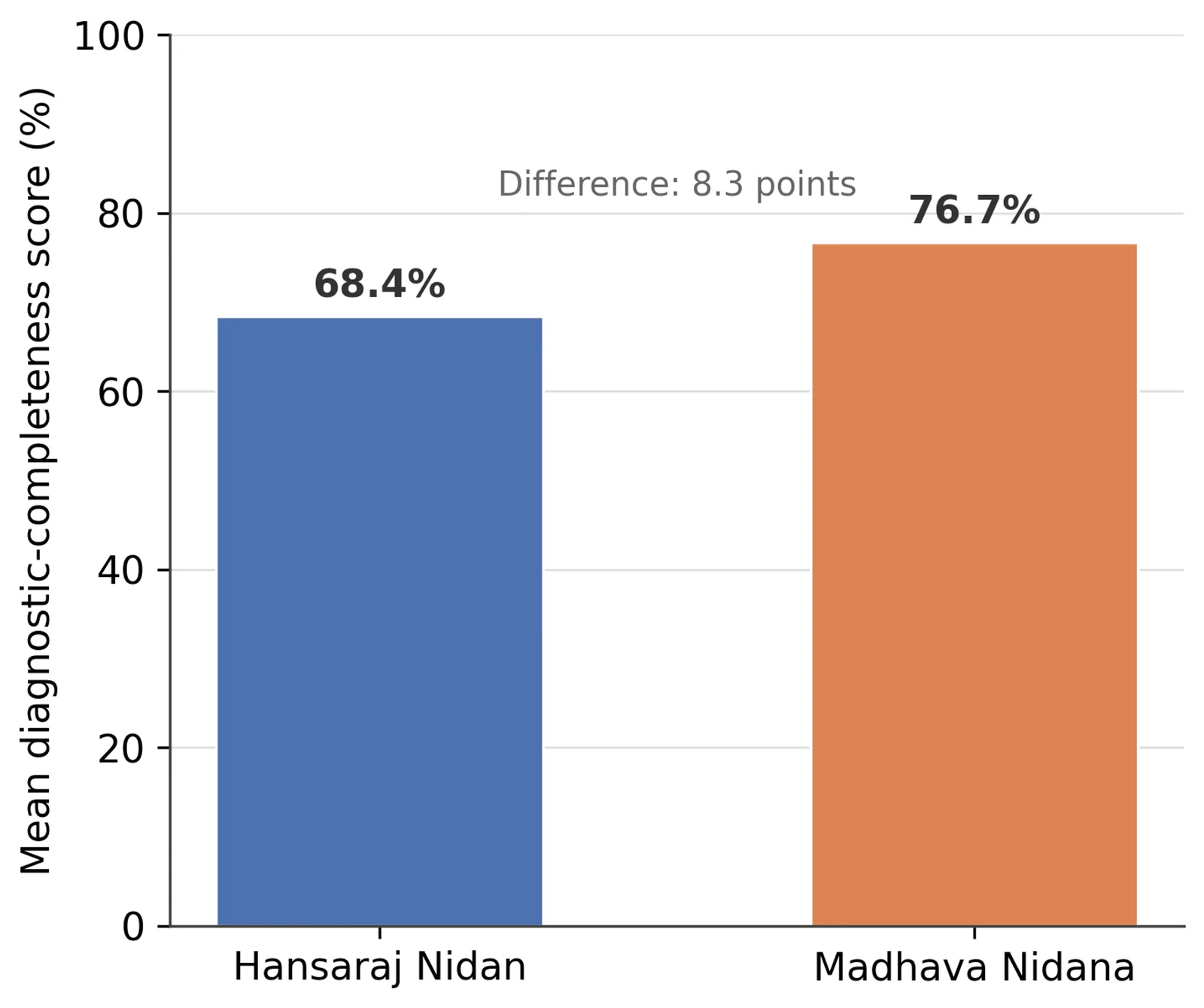
Comparative diagnostic-component documentation scores of Hansaraj Nidan and Madhava Nidana Image credit: Created by the authors using Microsoft Excel (Microsoft Corporation, Redmond, WA, USA)

Comparative interpretation

The comparison shows that Hansaraj Nidan and Madhava Nidana belong to the same broad Ayurvedic diagnostic tradition but differ in how they elaborate and organize the elements of disease evaluation. Both texts emphasize Nidana and Rupa, indicating that the delineation of causes and established clinical features formed the core of classical diagnosis. The more frequent documentation of Purvarupa, Samprapti, Upadrava, and prognosis in Madhava Nidana indicates fuller textual coverage of disease evolution and clinical outcome, in keeping with the combined role of the Nidana Panchaka in etiological assessment, early recognition, differential diagnosis, and therapeutic decision-making.

Madhava Nidana appears to have reorganized diagnostic material from earlier classical sources into a more accessible, disease-oriented format. Its recognition of specific disorders as independent clinical entities-Amavata (a disorder characterized by features associated with improperly digested metabolic material and joint involvement), for example-illustrates Madhavakara's refinement of Ayurvedic nosology through detailed clinical description [10]. Hansaraj Nidan drew on these earlier authorities but was not a mere restatement of Madhava Nidana: it further subdivides several disorders that gained prominence in later texts and practice, notably Jwara, age-related Hikka, Atishwasa, Udavarta, and certain ophthalmic diseases. Comparable patterns have been reported for other post-Madhava Nidana diagnostic works, which retained the overall structure of Madhava Nidana while adding diseases, omitting descriptions, rearranging chapters, and incorporating new diagnostic detail [9]. The overlap between the two texts thus reflects continuity of classical knowledge, while their unique and differently classified entities reflect the ongoing development of Ayurvedic diagnostic literature.

The limited documentation of Upashaya-Anupashaya in both texts may indicate a greater emphasis on etiology and symptomatology than on therapeutic response; nonetheless, Upashaya-Anupashaya remains a useful means of differentiating clinically similar diseases [11]. Comparative textual analysis also has educational value, clarifying synonyms, variations in diagnostic terminology, and the meaning of classical terms. Because textual interpretation depends on the edition, commentary, translation, and experience of the evaluator, standardized criteria and independent assessment are needed to improve its reliability [12].

Limitations

This study has several limitations. The analysis was based on selected printed editions and commentaries of each text, and readings vary across the surviving manuscripts and later recensions, so the counts reported here are specific to the editions examined. The mapping of diseases relied on scholarly interpretation of Sanskrit terminology and of synonymous or equivalent conditions, which introduces a degree of subjectivity, particularly for entities classified differently between the two texts. The completeness score treats each component as documented or not documented and does not grade the depth of description, and the quantitative findings are descriptive rather than inferential, as no formal statistical testing was performed. The diagnostic-completeness score reflects the presence of documented diagnostic components and does not capture the depth or qualitative detail of their description. Therefore, brief and extensively elaborated descriptions contribute equally to the score. Verse-level re-extraction, uniform diagnostic terminology, and independent expert verification by additional reviewers would further strengthen the reliability of the comparisons.

## Conclusions

Hansaraj Nidan and Madhava Nidana share a common Ayurvedic basis for disease classification and differ mainly in textual organization, diagnostic detail, terminology, and disease subdivision. In the records assessed, Madhava Nidana documented Purvarupa, Samprapti, complications, and prognosis more frequently, whereas Hansaraj Nidan continued this diagnostic tradition while adding distinctive variants of Jwara, Hikka, Shwasa, Udavarta, and ophthalmic disorders. The similarities point to continuity in Ayurvedic diagnostic knowledge, and the differences to the progressive development of classical nosology. Comparative mapping of the Nidana Panchaka, disease subtypes, complications, and prognostic categories can support textual interpretation, education, and clinical understanding. Because manuscripts, editions, and commentaries vary, the documentation differences reported here should be interpreted as specific to the editions examined and confirmed through verse-level verification, uniform terminology, and independent expert verification by additional reviewers.

## Appendices

**Table 6:** Original Sanskrit terms and their standardized clinical entity (English translation)

No.	Original Sanskrit term	Standardized clinical entity (English translation)	Broad clinical category
1	Jwara	Febrile disorders	Febrile (Jwara)
2	Vishama Jwara	Malarial/Intermittent fevers	Febrile (Jwara)
3	Dhatugata Jwara	Fevers localized in specific body tissues	Febrile (Jwara)
4	Sannipata Jwara	Severe acute fevers (involving all three doshas)	Febrile (Jwara)
5	Agantuja Jwara	Fevers of extrinsic origin (trauma/infection)	Febrile (Jwara)
6	Jirna Jwara	Chronic fevers	Febrile (Jwara)
7	Punaravartaka Jwara	Relapsing fevers	Febrile (Jwara)
8	Abhinyasa Jwara	Meningitis/encephalitis-like fevers	Febrile (Jwara)
9	Agnimandya	Dyspepsia/loss of appetite	Gastrointestinal
10	Ajeerna	Indigestion	Gastrointestinal
11	Arochaka	Anorexia	Gastrointestinal
12	Atisara	Diarrhea	Gastrointestinal
13	Grahani	Sprue syndrome/malabsorption	Gastrointestinal
14	Pravahika	Dysentery	Gastrointestinal
15	Arsha	Hemorrhoids	Gastrointestinal
16	Visuchika	Cholera/severe gastroenteritis	Gastrointestinal
17	Alasaka	Intestinal stasis/ileus	Gastrointestinal
18	Vilambika	Delayed digestion	Gastrointestinal
19	Krimi Roga	Helminthiasis/parasitic infections	Gastrointestinal
20	Chhardi	Vomiting/emesis	Gastrointestinal
21	Trishna	Polydipsia/excessive thirst	Gastrointestinal
22	Amlapitta	Hyperacidity/acid peptic disease	Gastrointestinal
23	Udara Roga	Ascites/generalized abdominal enlargement	Gastrointestinal
24	Pleeha Roga	Splenomegaly	Gastrointestinal
25	Yakrit Roga	Hepatomegaly/liver disorders	Gastrointestinal
26	Udavarta	Disorder of upward movement of bodily urges	Gastrointestinal
27	Anaha	Constipation with abdominal distension	Gastrointestinal
28	Shwasa / Atishwasa	Breathing disorders/excessive or labored breathing	Respiratory
29	Hikka	Hiccup (including age-related variants)	Respiratory
30	Kasa	Cough/bronchitis	Respiratory
31	Rajayakshma	Tuberculosis/phthisis	Respiratory
32	Urakshata	Chest trauma/pulmonary cavitation	Respiratory
33	Swarabheda	Hoarseness of voice	Respiratory
34	Peenasa	Chronic rhinitis/sinusitis	Respiratory
35	Hridroga	Ischemic and structural heart diseases	Cardiovascular
36	Pandu	Anemia	Cardiovascular/blood
37	Kamala	Jaundice/hepatic failure	Cardiovascular/blood
38	Halimaka	Advanced jaundice/chlorosis	Cardiovascular/blood
39	Raktapitta	Bleeding disorders/hemorrhagic diathesis	Cardiovascular/blood
40	Vata Vyadhi	Central and peripheral neurological disorders	Neurological
41	Apasmara	Epilepsy	Neurological
42	Unmada	Psychosis/insanity	Neurological
43	Murchha	Syncope/fainting	Neurological
44	Sanyasa	Coma	Neurological
45	Bhrama	Vertigo/dizziness	Neurological
46	Mutrakrichha	Dysuria	Urinary
47	Mutraghata	Urinary retention	Urinary
48	Ashmari	Urinary calculi/urolithiasis	Urinary
49	Mutrasada	Oliguria/renal impairment	Urinary
50	Basti Kundalika	Bladder atony/spasm	Urinary
51	Prameha	Metabolic urinary disorders (including diabetes)	Metabolic
52	Madhumeha	Diabetes mellitus	Metabolic
53	Medoroga (Sthaulya)	Obesity/dyslipidemia	Metabolic
54	Karshya	Severe emaciation/cachexia	Metabolic
55	Galaganda	Goiter	Metabolic
56	Prameha Pidaka	Diabetic carbuncles	Metabolic
57	Twak Roga (Kushtha)	Generalized skin diseases (including leprosy)	Dermatological
58	Shwitra	Vitiligo/leucoderma	Dermatological
59	Shitapitta	Urticaria	Dermatological
60	Udarda	Allergic rashes	Dermatological
61	Kotha	Erysipelas/herpes zoster	Dermatological
62	Visarpa	Erysipelas/herpes zoster	Dermatological
63	Masurika	Pox diseases (smallpox/chickenpox)	Dermatological
64	Romantika	Measles	Dermatological
65	Amavata	Rheumatoid arthritis (improper metabolism + joint involvement)	Musculoskeletal
66	Vatarakta	Gout/gouty arthritis	Musculoskeletal
67	Sandhigata Vata	Osteoarthritis	Musculoskeletal
68	Shula	Severe colic/spasmodic pain	Musculoskeletal/visceral
69	Parinama Shula	Peptic ulcer disease (pain related to digestion)	Musculoskeletal/visceral
70	Annadrava Shula	Gastric ulcer (continuous pain irrespective of food)	Musculoskeletal/visceral
71	Asrigdara (Pradara)	Menorrhagia/abnormal uterine bleeding	Reproductive (gynecological)
72	Yoni Vyapad	Structural and functional disorders of the female genital tract	Reproductive (gynecological)
73	Mudhagarbha	Obstructed labor/fetal malpresentation	Reproductive (obstetric)
74	Sutika Roga	Puerperal (postpartum) complications	Reproductive (obstetric)
75	Stanya Dosha	Disorders of breast milk	Pediatric/reproductive
76	Balaroga	General neonatal and pediatric diseases	Pediatric
77	Balagraha	Idiopathic pediatric infectious syndromes	Pediatric
78	Netra Roga	Ophthalmic disorders (includes distinctive variants)	ENT and Ophthalmic
79	Karna Roga	Otologic (ear) diseases	ENT and Ophthalmic
80	Nasa Roga	Rhinologic (nasal) diseases	ENT and Ophthalmic
81	Mukha Roga	Oral and dental diseases	ENT and Ophthalmic
82	Shiro Roga	Headaches and cranial diseases	ENT and Ophthalmic
83	Visha Roga	Acute poisoning and envenomation	Toxicological
84	Dushi Visha	Chronic, cumulative, or slow poisoning	Toxicological
85	Granthi	Benign tumors/cysts	Surgical/soft tissue
86	Arbuda	Malignant tumors	Surgical/soft tissue
87	Apachi	Chronic cervical lymphadenopathy	Surgical/soft tissue
88	Shlipada	Filariasis/elephantiasis	Surgical/soft tissue
89	Vidradhi	Internal and external abscesses	Surgical/soft tissue
90	Vrana	Chronic wounds and ulcers	Surgical/soft tissue
91	Bhagandara	Fistula-in-ano	Surgical/soft tissue

This document maps 91 standardized Ayurvedic disease entities to their original Sanskrit terms, combining explicitly provided data from the Hansaraj and Madhava Nidana analyses with standard historical Ayurvedic nosology.
